# Less Pollen-Mediated Gene Flow for More Signatures of Glacial Lineages: Congruent Evidence from Balsam Fir cpDNA and mtDNA for Multiple Refugia in Eastern and Central North America

**DOI:** 10.1371/journal.pone.0122815

**Published:** 2015-04-07

**Authors:** Benjamin Cinget, Sébastien Gérardi, Jean Beaulieu, Jean Bousquet

**Affiliations:** 1 Canada Research Chair in Forest and Environmental Genomics, Centre for Forest Research and Institute for Systems and Integrative Biology, Wood and Forest Sciences, Laval University, Québec, Québec, Canada; 2 Natural Resources Canada, Canadian Wood Fibre Centre, Canadian Wood Fibre Centre, Natural Resources Canada, Québec, Québec, Canada; National Cheng-Kung University, TAIWAN

## Abstract

The phylogeographic structure and postglacial history of balsam fir (*Abies balsamea*), a transcontinental North American boreal conifer, was inferred using mitochondrial DNA (mtDNA) and chloroplast DNA (cpDNA) markers. Genetic structure among 107 populations (mtDNA data) and 75 populations (cpDNA data) was analyzed using Bayesian and genetic distance approaches. Population differentiation was high for mtDNA (dispersed by seeds only), but also for cpDNA (dispersed by seeds and pollen), indicating that pollen gene flow is more restricted in balsam fir than in other boreal conifers. Low cpDNA gene flow in balsam fir may relate to low pollen production due to the inherent biology of the species and populations being decimated by recurrent spruce budworm epidemics, and/or to low dispersal of pollen grains due to their peculiar structural properties. Accordingly, a phylogeographic structure was detected using both mtDNA and cpDNA markers and population structure analyses supported the existence of at least five genetically distinct glacial lineages in central and eastern North America. Four of these would originate from glacial refugia located south of the Laurentide ice sheet, while the last one would have persisted in the northern Labrador region. As expected due to reduced pollen-mediated gene flow, congruence between the geographic distribution of mtDNA and cpDNA lineages was higher than in other North American conifers. However, concordance was not complete, reflecting that restricted but nonetheless detectable cpDNA gene flow among glacial lineages occurred during the Holocene. As a result, new cpDNA and mtDNA genome combinations indicative of cytoplasmic genome capture were observed.

## Introduction

During the last two decades, haploid cytoplasmic genomes have proven useful to infer phylogeographic structures of tree species because of their relative lack of recombination and small population size relative to nuclear genes [1]. These genomes also allow to compare the extent of seed and pollen gene flow in the Pinaceae [1], for which mitochondrial DNA (mtDNA) and chloroplast DNA (cpDNA) are maternally and paternally inherited, respectively [2,3]. MtDNA gene flow, which reflects seed dispersal in the Pinaceae, is generally more geographically restricted than cpDNA gene flow, which depicts both pollen and seed movements [4]. As a result, mtDNA polymorphisms generally allow for a better detection and delimitation of ancestral lineages than cpDNA markers [5], which generally show weak or no phylogeographic structures [6,7] owing to rapid homogenization of the genetic background of populations [5,8]. Nevertheless, when pollen-mediated gene flow is low, for instance because of geographic isolation or partial reproductive isolation between subspecific groups, cpDNA can provide valuable insights into intraspecific phylogeography (*e*.*g*. in North American conifers, *Picea glauca* [9]; *Picea mariana* [6]; *Pseudotsuga menziesii* [10]). Moreover, while the lack of intraspecific variation in conifer mtDNA genomes can represent an obvious obstacle to phylogeographical inference (*e*.*g*. [11]), polymorphism is usually easier to find in cpDNA, due to the availability of a set of highly polymorphic and transferable microsatellite markers ([12]). Thus, reliability of phylogeographic inferences is improved by surveying variation at both cytoplasmic genomes. This approach also allows to detect putative cytoplasmic capture events (*e*.*g*. at the intraspecific level, [6,10]), which can lead to erroneous phylogeographic inferences when overlooked.

Geographic distribution and genetic structure of North American boreal trees have been largely shaped by vicariance events caused by Pleistocene climatic oscillations (*e*.*g*. [1,13,14]). The expansion of ice sheets during the last glacial cycle triggered southward population migration, generally associated with population size reduction. As the glaciers receded, populations recolonized newly available habitats, expanding northward during the Holocene [4,15,16]. Two survival strategies were then possible during the last glacial maximum (LGM, 20 kyr BP): migrating southward to follow the progression of the ice front and/or surviving in various isolated refugia of smaller effective population size [16–18].

The combined use of fossil [17,18] and genetic data [6,7,10,19–22] brought insights regarding the putative location of several of these refugia in North America. In the eastern part of the continent, molecular evidence pointed to the persistence of species in isolated cryptic refugia after their southward retreat. Some of these studies (*e*.*g*. *Setophaga ruticilla* [23]; *Picea mariana* [6,19]) suggested such a putative refugium in Labrador, a controversial hypothesis first proposed by Tremblay & Schoen in 1999 [24]. However, its exact location remains uncertain given the absence of a reliable paleorecord in this region. The very existence of ice-free areas in this region during LGM remains a long-standing debate (*e*.*g*. [25–27]). A second putative cryptic refugium in close proximity to the southeastern margin of the Laurentide Ice Sheet was proposed in coastal areas of the Maritimes for conifer species (*Pinus banksiana* [7]; *Pinus resinosa* [28]). Finally, several trees (*Fagus grandifolia*, *Acer rubrum* [29]) and animal species (*Melanoplus spp*. [30]; *Tamias striatus* [31]; *Nigronia serricornis* [32]) are thought to have persisted in small populations in close proximity to the margin of the Laurentide Ice Sheet in the Great Lakes area [33]. Species able to survive in such cryptic refugia may have been favoured during the first stage of postglacial colonization [7,34,35], but more intraspecific evidence for genetically differentiated glacial populations in these areas is needed to support this hypothesis.

Balsam fir [*Abies balsamea* (L.) Mill.] has a continuous longitudinal distribution ranging from Labrador to central Alberta, while its latitudinal distribution extends between northern Québec and south Wisconsin [36,37]. It occurs throughout the Canadian temperate and boreal forests, but does not grow as far north as other boreal conifers such as *Picea mariana*, *Picea glauca* or *Larix laricina* [38]. Nevertheless, the species has a great capacity to colonize newly available or disturbed habitats in association with white spruce, especially in the northern part of its natural range [39,40]. Contrary to many other conifers, balsam fir has typically low pollen production [41] and short pollen dispersal distance, presumably owing to the large size of its grains (> 80 μm) [42–44] and high total velocity [45]. Thus, such pollen singularities may translate into lower cpDNA gene flow and stronger cpDNA population structure than typically observed in other sympatric conifers. If confirmed, such limited cpDNA gene flow should also result in increased congruence between cpDNA and mtDNA population structure, which should help to delimitate genetically distinct glacial lineages.

The main objective of this study was to infer the population structure of balsam fir from mtDNA and cpDNA variation and take advantage of the species low pollen production and short pollen dispersal distance to gain insights into large-scale phylogeographic patterns in North America. In balsam fir, highly polymorphic cpDNA microsatellites (or cpSSR) are expected to reveal a stronger population structure than typically observed in other widespread boreal conifers, which should also translate into increased congruence between cpDNA and mtDNA geographical structures. Thus, given the wide distribution of the species from central Canada to the Atlantic Ocean, investigating its phylogeography may bring new insights regarding the existence of several cryptic or controversial glacial refugia in eastern North America.

## Materials and Methods

### Ethics Statement

The three fir species sampled in this study (*Abies balsamea, Abies lasiocarpa* and *Abies fraseri*) are neither endangered or protected according to the 'Endangered & Threatened Species List' provided by the U.S. Fish & Wildlife Service (U.S.A.) or the 'List of Wildlife Species at Risk' provided by Environment Canada (Canada). However, Fraser fir appears endangered according to the IUCN Red List of Threatened Species. No permit was required to collect tissue in any location sampled in this study. Samples were either collected on public lands or in U.S.A. State Parks or Canadian Provincial Parks after permission to do so was granted by park managers. All seed samples were obtained from collections of the National Tree Seed Centre (Canada, contact: Mr. Bernard Daigle). Live twigs were collected in a non-destructive and non-disruptive manner, so as to not interfere with the growth and/or health of either sampled species.

### Population sampling and DNA extraction

In total, 1616 samples from 107 balsam fir populations covering the natural range of the species were collected with an average of 15 samples per population. Twigs were collected for 99 populations and collections of the National Tree Seed Centre provided bulk seed samples for eight additional balsam fir natural populations from Newfoundland and Prince Edward Island (from a minimum of 10 mother trees per population, S1 Table).

**Fig 1:**
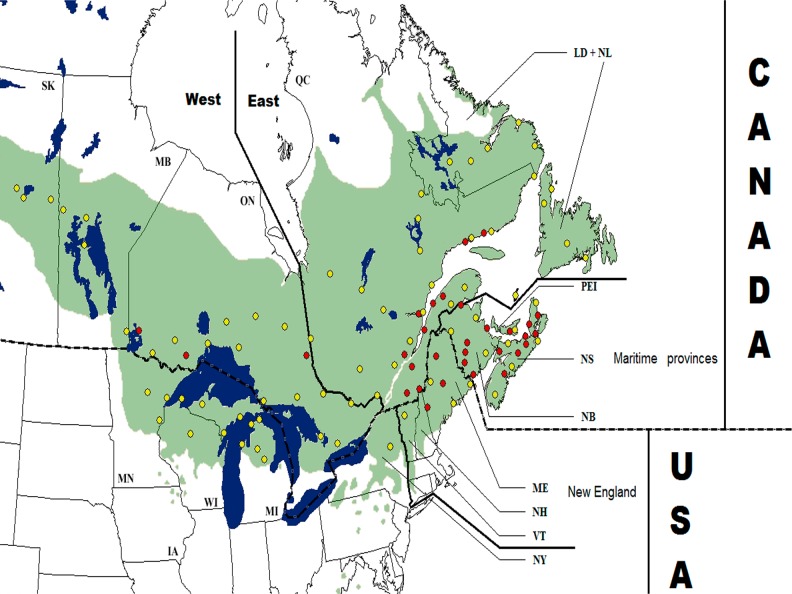
Distribution of the 127 populations sampled across the natural range of *Abies balsamea* (green). All population (yellow and red circles) were used for the mtDNA study, while only populations represented by a yellow circle were considered for the cpDNA study. Delimitation of geographic regions, states and provinces mentioned in the text are illustrated. Abbreviations: SK = Saskatchewan, MB = Manitoba, ON = Ontario, QC = Québec, NL+LD = Newfoundland & Labrador, NS = Nova Scotia, NB = New Brunswick, PEI = Prince Edward Island, MN = Minnesota, WI = Wisconsin, MI = Michigan, NY = New-York, VT = Vermont, NH = New-Hampshire, ME = Maine.

For needle samples, DNA was extracted from 0.05 g of vegetal material using the DNeasy 96 plant kit (Qiagen, Mississauga, Ontario, Canada) and following the manufacturer’s instructions. Seeds were dissected to isolate megagametophytes, which cytoplasmic genetic background is representative of mother trees, and total DNA was extracted from megagametophytes with the DNeasy 96 plant kit (Qiagen, Mississauga, Ontario, Canada). Two population sets were analysed. The first group, that contained all populations (107) and all trees sampled (1616), was used to analyze mtDNA population structure (see next section for details regarding the screening of polymorphism). Because cpDNA data provides less insights than mtDNA data regarding the fine scale historical population structure of boreal conifers [6,7,9,22], cpDNA data analyses were conducted on a reduced set of populations. This population subset included 955 trees from 75 populations (at a rate of 12 individuals per population) separated by at least 200 km from each other (Fig 1).

Two additional subsets included three populations of *Abies lasiocarpa* (subalpine fir, 24 individuals) and three populations of *Abies fraseri* (Fraser fir, 19 individuals), two species phylogenetically closely-related to *A*. *balsamea* [46–48]. They were primarily used to make inferences regarding the ancestral or derived nature of haplotypes and phylogenetic relationships among balsam fir lineage (see below). Balsam fir populations sampled were located more than 1000 km away from the easternmost part of *A*. *lasiocarpa*’s natural range in western Alberta, and from the natural range of *A*. *fraseri*, which is endemic to the southern Appalachian Mountains in eastern U.S.A.

### Screening of mtDNA polymorphism, PCR conditions and genotyping

A total of 43 regions of the mitochondrial genome were screened for polymorphism using a panel of 27 individuals of *A*. *balsamea* from 9 geographically remote populations (see S2 Table for details). Polymorphism was only found in the fourth intron of the mitochondrial *nad* 5 gene (*nad5-4*). Using primers developed by Wu *et al*., 1998 [49], sequences were obtained for 36 *A*. *balsamea* individuals sampled from 12 populations distributed throughout the species’ range. In addition, three populations of *A*. *fraseri* (3 individuals per population) and *A*. *lasiocarpa* (3 individuals per population) were also sequenced. These sequences were used to determine the nature of polymorphism and to design new internal primers using OligoPerfect Designer (Invitrogen, Life Technologies Corporation, Cleveland, Ohio, USA) for further sequencing. These internal primers (named *nad*5-4*Ab*, see S2 Table) were then used to amplify DNA from all samples.

DNA was amplified in a PTC-225 thermal cycler (Bio-Rad, Mississauga, Ontario, Canada) using 25 to 50 ng of DNA template, 0.1 μM of each primer, 0.1 mM of each dNTP, 1X of reaction buffer, 1.5 mM MgCl_2_ and 0.125 units of Platinum *Taq* DNA polymerase (Invitrogen, Carlsbad, California, USA). Polymerase chain reaction (PCR) conditions consisted of an initial denaturation step (4 min at 94°C), followed by 35 cycles of denaturation (1 min at 94°C), annealing (30 s at 56.3°C), extension (1 min at 72°C), and a final extension (10 min at 72°C). PCR products were sequenced on an ABI-3730xl DNA Analyzer (Applied Biosystems, Life Technologies Corporation, Cleveland, Ohio, USA) using the dideoxynucleotide chain termination procedure (Sanger method). Sequence alignment and allele scoring were performed using the CodonCode Aligner version 3.7.1 software (CodonCode Corporation, Centerville, Massachusetts, USA).

### Screening of cpDNA polymorphism, PCR conditions and genotyping

Microsatellite markers designed by Vendramin *et al*., 1996 [12] were used to infer cpDNA population structure in balsam fir. Polymerase chain reaction (PCR) was conducted in a PTC-225 thermal cycler (Bio-Rad, Mississauga, Ontario, Canada) with 10 ng of DNA template, 20 μM of each primer, 10 mM of each dNTP, 1X of reaction buffer, 1.5 mM MgCl_2,_ 20 μM of fluorescent-labelled M13 primer (M13R/IRD800, MWG-Biotech, Huntsville, Alabama, USA), and 0.05 unit of Platinum *Taq* DNA polymerase (Invitrogen, Carlsbad, California, USA). Amplifications were performed according to the protocol described in Oetting *et al*., 1995 [50]. An additional tail of 19 pb, identical to M13 forward primer (5’-CACGACGTTGTAAAACGAC-3’), was added to the 5’end of forward primers. PCR conditions were the following: initial denaturation (3 min at 94°C) followed by 35 cycles of denaturation (1 min at 94°C), annealing (1 min at 56.3°C), extension (1 min at 72°C), and a final extension (10 min at 72°C). Amplification products and IRDye fluorescent size standards (LI-COR, Lincoln, Nebraska, USA) were loaded in 8% denaturing polyacrylamide gels and analyzed on a LI-COR 4200 sequencer (LI-COR, Lincoln, Nebraska USA) to detect length variations. Out of the 20 primer pairs tested on a panel of 24 populations, four (*Pt*26081, *Pt*30204, *Pt*63718 and *Pt*71936) revealed intraspecific length polymorphism. However, only two of them (*Pt*30204 and *Pt*71936) showed consistent amplifications and therefore, were retained for genotyping.

The two cpDNA microsatellite markers (*Pt30204* and *Pt71936*) were further sequenced for a subset of 81 individuals (three individuals for each variant, see Results section) in order to detect putative homoplasy of fragment length. Sequencing was performed with a Sequenase GC-rich kit (Applied Biosystems, Cleveland, Ohio, USA) using the dideoxynucleotide chain termination procedure on an ABI 3130xl genetic analyzer (Applied Biosystems, Cleveland, Ohio, USA).

### Data analysis

Observed numbers of mitotypes and chlorotypes (*nh*_mt_ and *nh*_cp_), as well as mitotype and chlorotype diversity (*H*_mt_ and *H*_cp_, equivalent to the expected heterozygosity, *H*_e_, for diploid data; [51]) were calculated for each population. Evolutionary relationships among mitotypes were investigated with a minimum-spanning tree using the software TCS [52] with a ‘fix connection limit’ set at five steps.

Population fixation indices based on allele size and allele identity (*G*_STcp_, *R*_STcp_ for cpDNA and *G*_STmt_, *N*_STmt_ for mtDNA) were then estimated with Permut/cpSSR [53] and the presence of a phylogeographic structure was tested with 10 000 permutations. Contrary to *G*_ST,_ *N*_ST_ and *R*_ST_ take into account the relatedness among haplotypes to estimate population differentiation. Thus, a significantly higher value for *N*_ST_ or *R*_ST_ than for *G*_ST_ would be indicative of a phylogeographical structure. Jost’s differentiation index (*D*, [54]) was also calculated from mitotypes and chlorotypes because unlike *G*_ST_, this measure is independent of gene diversity [55]. Hence, this index is useful to compare population differentiation estimates obtained from markers with heterogeneous levels of polymorphism and different mutation rates [54,55], such as mtDNA sequence indels and cpDNA SSRs, as used in the present study. Jost’s differentiation estimates (*D*_mt(group)_ and *D*_cp(group)_) were also calculated among mtDNA and cpDNA BAPS groups (see below for groups delineation) in order to compare the magnitude of genetic differentiation among mtDNA and cpDNA lineages.

Population structure was assessed independently for mtDNA and cpDNA data, using the ‘spatial clustering of groups’ option implemented in the software BAPS 5.4 [56]. BAPS allocates populations in a user-defined number of groups (*k*-value) so as to maximize the differentiation among groups using *k*-values and allele frequencies as varying parameters. A logarithm value of maximal likelihood (log (ml) associated with the 10 best partitions visited is estimated. However, the recommended approach to determine the optimal partition [56] yielded an overly larger number of groups, far exceeding the number of putative North American refugia. Both the spatial distribution of groups and the genetic background of populations within groups suggested that several groups corresponded to suture zones between lineages (S1 and S2 Figs), as already observed in black spruce [57]. Therefore, an alternative method was used to determine the optimal partition. The ‘Fixed K’ mode [56–58] was enabled and 100 runs were computed for each *k*-value ranging between 2 and 10. The resulting optimal log (ml) values were plotted as a function of the number of clusters and the number of groups corresponding to the inflexion point of the curve was considered optimal (S1 Fig). Additionally, for each optimal cpDNA and mtDNA partition obtained with BAPS, relationships among groups of populations were assessed by constructing a neighbor-joining dendrogram (NJ) [59] using the chord distance. This genetic distance was chosen for its independence from the underlying mutation model [60]. The three populations of *A*. *fraseri* (19 individuals) were used as outgroups in order to root the trees. Trees were generated using the software MEGA 4 [61].

A hierarchical analysis of molecular variance (AMOVA) was conducted with Arlequin 3.1 [62] to assess the partitioning of genetic variation within populations, among populations within BAPS groups and among BAPS groups, for mtDNA and cpDNA data independently. Inherently, this method also allowed to assess the relative contribution of different evolutionary processes to overall population differentiation. Since *F*_CT_ estimates the level of genetic differentiation among groups of populations presumably representative of genetically distinct lineages, this index reflects historical genetic differentiation due to geographic isolation of populations in glacial refugia. Contrastingly, *F*_SC_ estimates population differentiation within historical lineages. Thus, this index rather reflects seeds and pollen dispersal abilities over generations and their homogenizing effect on genetic diversity (*i*.*e*. mtDNA and cpDNA gene flow) rather than historical divergence among lineages. The statistical significance of differentiation indices was tested using 50 000 permutations. Estimates of population differentiation within mtDNA and cpDNA BAPS groups (*F*_SCmt_ and *F*_SCcp_) were then used to estimate the effective number of migrants per generation (*N*_e_ *m*_mt_ and *N*_e_ *m*_cp_ for seeds and pollen, respectively) according to Takahata & Palumbi, 1985 [63], and thereby, assess the extent of contemporary seed and pollen gene flow in *A*. *balsamea*. For the sake of comparison, similar estimates were derived for *Picea mariana*, *Pinus banksiana* and *Tsuga canadensis* from previously published cytoplasmic marker data [6,7,22].

## Results

### MtDNA polymorphisms

Out of the 43 mitochondrial regions initially screened, *nad*5-4 was the only polymorphic locus. This result was expected given the much conserved nature of plant and conifer mtDNA exons and introns [64,65] and little mtDNA polymorphism observed among closely-related mesoamerican firs [66]. Sequence variation was found in two distinct regions of *nad*5-4 which, once combined, yielded five mitochondrial haplotypes or mitotypes (Table 1). No species-specific variant was detected within the two closely-related fir species tested (*A*. *fraseri* and *A*. *lasiocarpa*). All *A*. *fraseri* and *A*. *lasiocarpa* individuals harbored mitotype I and II, respectively, while these two mitotypes were also observed in *A*. *balsamea* (GenBank accession nos. KJ705284-KJ052288).

**Table 1:** Description of the five variants (mitotypes) detected in the intron 4 of the *nad5* mtDNA gene of *Abies balsamea*. *Abies fraseri* and *Abies lasiocarpa*, two phylogenetically closely-related species to *A*. *balsamea*, were fixed for mitotype I and mitotype 2, respectively.

	*nad* 5 intron 4		
	Poly. 1*		Poly. 2*
Mitotypes	122–137**		170–177**
I	GATATATAGATATATA	GATAGATATATAGATAGATAGATAGATAGATA	GATAGATA
II	GATATATAGATATATA	GATAGATATATAGATAGATAGATAGATAGATA	GATA----
III	GATATATA--------	GATAGATATATAGATAGATAGATAGATAGATA	GATAGATA
IV	GATAGATA--------	GATAGATATATAGATAGATAGATAGATAGATA	GATAGATA
V	GATA------------	GATAGATATATAGATAGATAGATAGATAGATA	GATA----

* Poly, Polymorphic region;

** numbers indicate nucleotide positions in the longest sequence obtained with *nad*5-4*Ab* primers (see Materials and Methods for more information). Dashes indicate alignment gaps.

**Fig 2:**
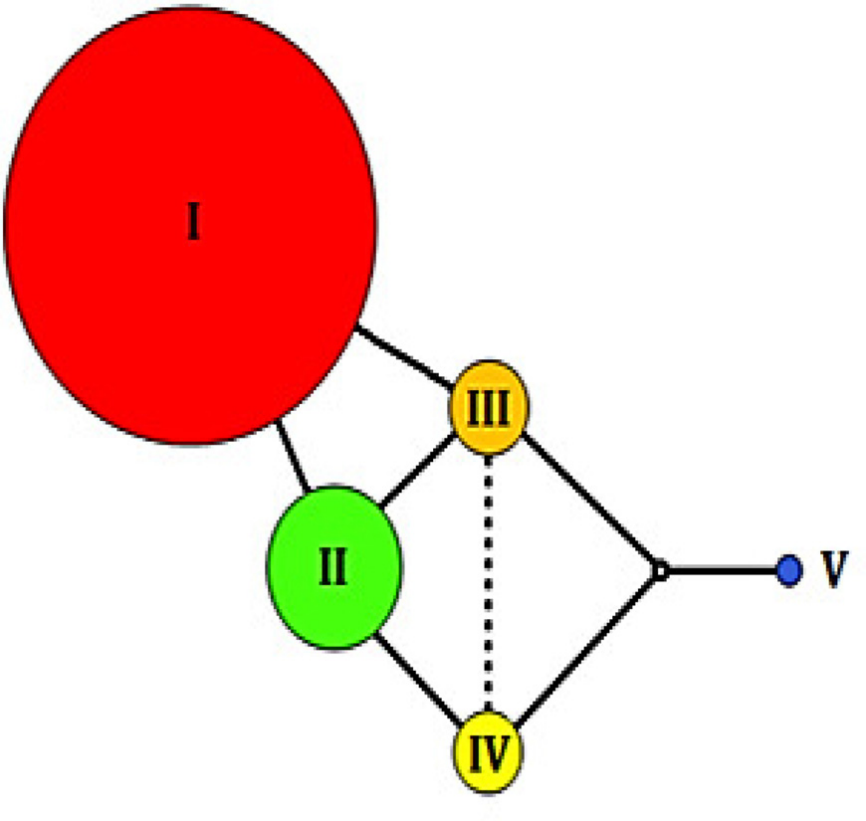
Haplotype network of the five mtDNA haplotypes observed in *Abies balsamea*. The size of the circles is proportional to the overall relative frequency of each haplotype in natural populations (See S1 Table for exact frequencies). The “**o**” symbol represents intermediate haplotype not found in the sample. The dotted line represents a putative single mutational step between mitotypes III and IV (see Results section for more information). Mitotype I wasfixed for *Abies fraseri* individuals and mitotype II for *Abies lasiocarpa* individuals.

**Fig 3:**
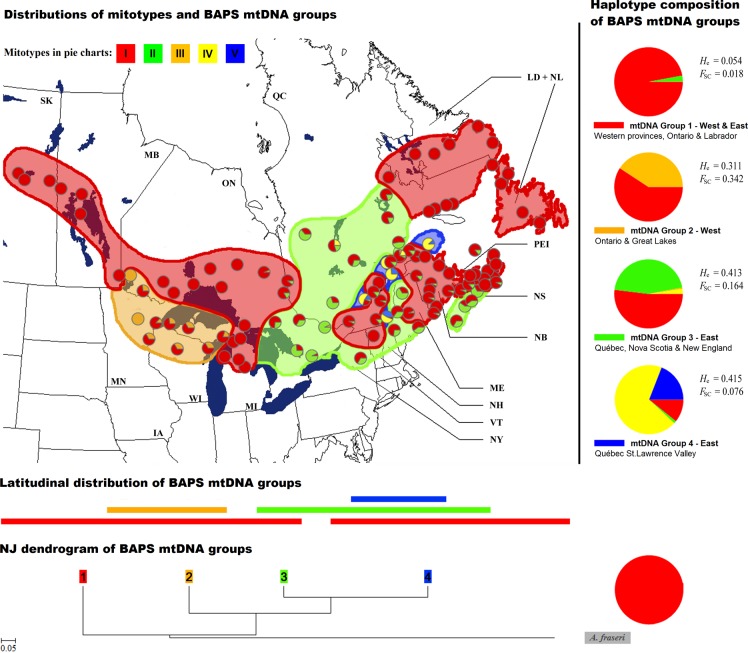
Geographical distribution of mtDNA haplotypes for 107 populations of *Abies balsamea*. Groups of populations genetically homogeneous determined by the Bayesian analysis of population structure (BAPS) are represented in colored areas and mitotype composition of each group is illustrated on the right (haplotype colors correspond to those of Fig 2). Neighbor-Joining relationships among BAPS mtDNA groups based on chord distances are depicted at the bottom of the figure (group colors correspond to those of the map and the length of horizontal bars represents the longitudinal spread of each group). See Fig 1 for abbreviations codes.

Mitotype I was predominant in balsam fir with around 80% of the individuals bearing this variant (Fig 2). It was also the most widely distributed (Fig 3). Mitotype II was the second most abundant (frequency, *f* = 0.112), but was geographically restricted to the southeastern part of the species’ natural range. All three other mitotypes (III, IV and V) were more locally distributed and less frequent (*f* = 0.040, *f* = 0.044 and *f* = 0.010, respectively). Both average mitotype diversity (*H*_mt_) and average mitotype number (*nh*_mt_) were low with values of 0.168 and 1.6, respectively (S1 Table).

### CpDNA polymorphisms

The sequencing of 81 trees confirmed that SSRs *Pt*30204 and *Pt*71936 presented the same polymorphisms as those previously reported for these two loci [67]. The length polymorphisms observed at loci *Pt*30204 and *Pt*71936 were due to variation in repeat number. As expected, polymorphisms were caused by mononucleotide repeats, (A)_*n*_ followed by (T)_*n*_ for *Pt*30204, and (A)_*n*_ for *Pt*71936. Although *Pt30204* included two variable regions, no evidence of fragment length homoplasy was found in the sequences analyzed.

**Fig 4:**
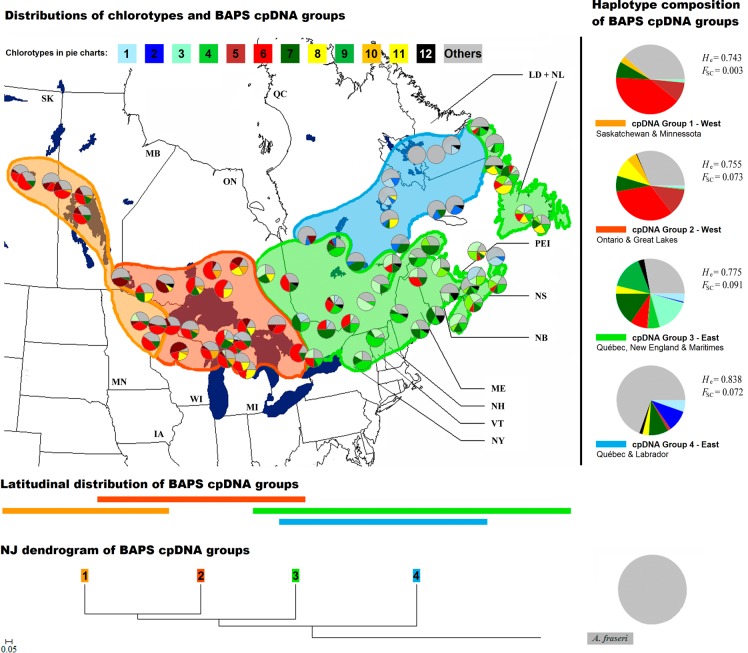
Geographical distribution of the 11 most frequent cpDNA haplotypes in 75 populations of *Abies balsamea*. Groups of populations genetically homogeneous determined by the Bayesian analysis of population structure (BAPS) based on all chlorotypes are represented in colored areas and the chlorotype composition of each group is illustrated on the right. Neighnor-Joining relationships among BAPS cpDNA groups based on chord distances are depicted at the bottom of the figure (group colors correspond to those of the map and the length of horizontal bars represents the longitudinal spread of each group). See Fig 1 for abbreviations codes.

For the 955 trees surveyed, a total of 15 and 12 alleles were found at loci *Pt*30204 and *Pt*71936, respectively. When considered together, the two loci yielded a total of 86 chlorotypes, from which 11 had a frequency greater than 0.015 (Fig 4). Among these, chlorotypes 6 and 7 were the most abundant (*f* = 0.184 and 0.111, respectively), and chlorotype 11 was the less frequent (*f* = 0.016). Most of the variants (76%) were rare chlorotypes (*f* < 0.01) and one third of those were private (population-specific). Estimates of average chlorotype diversity (*H*_cp_ = 0.773) and average number of chlorotypes (*nh*_cp_ = 6.7) were much higher than those obtained for mtDNA data (S1 Table).

### Distribution of mitotypes, mtDNA differentiation and population structure

Overall mtDNA differentiation among populations and among BAPS mtDNA groups was significant (*F*_STmt_ = 0.688 and *F*_CTmt_ = 0.599, *P* < 0.0001; Table 2), but population differentiation was low compared with that of other conifers (see Discussion). It appeared even lower when estimated using Jost’s index (*D*_mt_ = 0.202 and *D*_mt(group)_ = 0.552). Population differentiation within BAPS mtDNA groups (see below) followed the same trend (*F*_SCmt_ = 0.220, *P* < 0.0001; Table 2) and estimates of mtDNA migration (*N*_e_ *m*_mt_) among populations within BAPS groups indicated that seed gene flow was low (*N*_e_ *m*_mt_ = 1.771). The distribution of mitotypes was well geographically and phylogeographically structured (*G*_STmt_ = 0.480 < *N*_STmt_ = 0.662; *P* < 0.01) and differentiation among mtDNA groups was high and significant (*F*_CTmt_ = 0.599, *P* < 0.0001; Table 2).

**Table 2:** Hierarchical analysis of molecular variance (AMOVA) based on chlorotype and mitotype frequencies for populations of *Abies balsamea* grouped according to mtDNA and cpDNA population structures inferred with BAPS.

Source of variation	Df^1^	SS^2^	VC^3^	Variation (%)	*F*-statistics^4^
**mtDNA**					
Among mtDNA groups	3	857.1	1.001	59.9	*F*_CT_ = 0.599*
Among populations within groups	103	282.2	0.147	8.9	*F*_SC_ = 0.220*
Within populations	1505	785.2	0.522	31.2	*F*_ST_ = 0.688*
Total	1611	1924.5	1.670		
**cpDNA**					
Among cpDNA groups	3	44.9	0.062	7.8	*F*_CT_ = 0.078*
Among populations within groups	71	97.9	0.055	6.9	*F*_SC_ = 0.075*
Within populations	880	598.8	0.680	85.4	*F*_ST_ = 0.146*
Total	954	741.5	0.797		

^1^Df, degrees of freedom;

^2^SS, sum of squares;

^3^VC, variance component;

^4^ *F*_CT_, differentiation among groups;

*F*_SC_, differentiation among populations within groups;

*F*_ST_, differentiation among populations;

*P < 0.0001.

With an optimal partition obtained for *k*-value = 4 (see S3 Fig), the Bayesian analysis of population structure resulted in four genetically homogeneous mtDNA groups (Fig 3). The first mtDNA group included populations carrying mitotype I at high frequency. This group was divided into two geographically disjunct subgroups: 1) populations distributed between Saskatchewan and Ontario (western part) and 2) populations scattered across northern New-England, northeastern Québec, Newfoundland and Labrador (eastern part). The second mtDNA group contained populations from the western Great Lakes region, in which mitotypes I and III co-occurred. The mtDNA group #3 included populations characterized by the predominance of mitotype II. They were mainly located in eastern Ontario, central Québec, the Maritimes and in northeastern United States (the southernmost part of balsam fir natural range). Finally, the mtDNA group #4 encompassed populations essentially located in the St. Lawrence River Valley and carrying mitotypes IV and V.

According to the NJ dendrogram, mtDNA group #1 was the most ancestral (in red on Fig 3). All populations included in this group carried a large proportion of mitotype I (between 86 and 100%), which was also the only mitotype found in the outgroup (*A*. *fraseri*). MtDNA group #2 (in orange on Fig 3), characterized by the predominance of mitotypes I and III, was the second most ancestral followed by mtDNA groups #3 and #4, which were more closely-related (in green and blue on Fig 3). Altogether, genetic divergences represented in the NJ dendrogram seemed primarily attributable to differences in the frequency of mitotype I within each mtDNA group.

### Distribution of chlorotypes, cpDNA differentiation and population structure

As expected, cpDNA differentiation among populations and among BAPS cpDNA groups was significant and lower than that estimated with mitotypes (*F*_STcp_ = 0.146, *F*_CTcp_ = 0.078, *P* < 0.0001; Table 2). However, differentiation among populations and BAPS cpDNA groups appeared much higher according to Jost’s estimate (*D*_cp_ = 0.610 and *D*_cp(group)_ = 0.594, respectively). More noticeably, and contrary to expectations, Jost’s estimates revealed that cpDNA population differentiation was higher than mtDNA population differentiation (*D*_cp_ = 0.610 > *D*_mt_ = 0.202) and that differentiation among BAPS groups was comparable between mtDNA and cpDNA data (*D*_(group)_ = 0. 552 and 0.594 for mtDNA and cpDNA, respectively). Population differentiation within cpDNA BAPS groups likely representative of different glacial lineages (see next paragraph and Discussion) also appeared high (*F*_SCcp_ = 0.220, *P* < 0.0001; Table 2). Although higher than mtDNA gene flow, cpDNA gene flow was lower in balsam fir (*N*_e_ *m*_cp_ = 6.20) than in the other sympatric conifers previously surveyed (see Discussion). Similarly to mtDNA, differentiation among cpDNA BAPS groups was high and significant (*F*_CTcp_ = 0.078, *P* < 0.0001; Table 2), while the comparison of *G*_STcp_ and *R*_STcp_ indicated the presence of a significant phylogeographic structure (*G*_STcp_ = 0.104 < *R*_STcp_ = 0.272; *P* < 0.01).

The Bayesian analysis of cpDNA population structure revealed four genetically distinct population groups (Fig 4), the best partition being obtained for *k*-value = 4 (see S1 Fig). The two western groups (cpDNA groups #1 and #2) were characterized by populations carrying chlorotype 6 at high frequency. Populations from Saskatchewan, Manitoba and Minnesota were assigned to cpDNA group #1, while populations from Ontario, Michigan, and Wisconsin were included in cpDNA group #2. These two groups differed essentially from each other by the presence of chlorotype 8 in cpDNA group #2. The cpDNA group #3 was composed of populations from the eastern part of balsam fir natural range (eastern Ontario, southern Québec, and Newfoundland). They were characterized by the predominance of chlorotypes 3, 4 and 9. Finally, the cpDNA group #4 included all northeasternmost populations (northern Québec and Labrador) other than populations from Newfoundland. This last cpDNA group was characterized by a large proportion of rare alleles (0.69), a high frequency of chlorotype 2 (0.10), and the complete absence of the most frequent variant (chlorotype 6, Fig 4).

The cpDNA BAPS group #4 (in blue on Fig 4) was located at the most basal position on the NJ dendrogram and presented obvious genetic proximity with *A*. *fraseri* (Fig 4). CpDNA groups #1 and #2 (in orange and red on Fig 4, western Canada and the Great Lakes) were more related to each other than to other groups, and were both located in the western part of the natural range. CpDNA group #3 (in green on Fig 4, populations from southern Québec and the Maritimes) had an intermediate position in the NJ dendrogram, a position that was also reflected in its geographic distribution (Fig 4).

## Discussion

### Cytoplasmic diversity and population differentiation trends

Balsam fir mitotype diversity (*H*_mt_ = 0.166) was similar to that of the sympatric and largely distributed boreal species black spruce (*Picea mariana*, 0.191 [30]). Within the genus *Abies*, three Japanese species (*Abies firma*, *Abies sachalinensis* and *Abies homolepis*) harbour reportedly higher mtDNA diversity than *A*. *balsamea* [68]. Low mtDNA diversity in *A*. *balsamea* is possibly related to the fixation of mitotype I in a large number of populations of presumably distinct glacial origin. This could indicate that genetic drift during isolation in refugia or early Holocene recolonization depleted mtDNA diversity in some balsam fir glacial lineages.

Mean cpSSR diversity in balsam fir (*H*_cp_ = 0.773) was in the same range as that observed in *A*. *fraseri* (*H*_cp_ = 0.84 [67]), *Abies alba* (*H*_cp_ = 0.98 and 0.96 [69,70]), *Abies nordmanniana* (*H*_cp_ = 0.98 [71]), *Abies nebrodencis*, *Abies numidica*, *Abies cephalonica* (*H*_cp_ = 0.846; 0.968; 0.993, respectively [70]), *Abies sibirica*, *Abies nephrolepis*, *Abies sachalinensis*, *A*. *holophylla* (*H*_cp_ = 0.87, 0.94, 0.96, and 0.91, respectively [72]) and in Mesoamerican *Abies* [66]: *Abies flinckii* (*H*_cp_ = 0.802), *Abies guatemalensis* (*H*_cp_ = 0.934), *Abies hickelii* (*H*_cp_ = 0.937), and *Abies religiosa* (*H*_cp_ = 0.908). CpSSR diversity in balsam fir was also comparable to that of other North American boreal and temperate conifers: for instance, black spruce (*P*. *mariana*, *H*_cp_ = 0.80 [6]), white spruce (*Picea glauca*, *H*_cp_ = 0.803; Gérardi & Bousquet, unpublished data), jack pine (*Pinus banksiana*, *H*_cp_ = 0.780 [7]) or eastern hemlock (*Tsuga canadensis*, *H*_cp_ = 0.727 [22]).

MtDNA differentiation among populations (*G*_STmt_ = 0.480) was significant but lower than that observed for other widely distributed North American boreal conifers sympatric with balsam fir (Table 3). The difference was even more pronounced when Jost’s estimates were compared (*D*_mt_ = 0.202 and 0.537 for *A*. *balsamea* and *P*. *mariana*, respectively). However, mtDNA population differentiation was possibly underestimated in balsam fir due to the fact that the mtDNA group #1 included populations of diverse ancestry which shared the same mtDNA background (see glacial lineage delineation for further details). This inference is all the more likely that the mutation rate of mtDNA in plants and conifers is low [65,66], and given that these populations belong to several genetically distinct groups based on the analysis of cpDNA variation. MtDNA population differentiation in *A*. *balsamea* was also lower than that of Mesoamerican firs [66] (Table 3). However, these species typically occur in small high altitude populations which experience strong genetic drift and very limited mtDNA gene flow due to geographic isolation [66].

**Table 3:** Genetic differentiation estimates for cpDNA and mtDNA across various *Abies* species and other conifers sympatric to *Abies balsamea*.

	mtDNA			cpDNA			
Species	*G*_ST_ ^1^	*F*_SC_ ^2^	*D* ^*3*^	*G*_ST_ ^1^	*F*_SC_ ^2^	*D* ^*3*^	Reference
***Abies balsamea***	0.480	0.220	0.202	0.104	0.075	0.610	This study
***Abies***							
*A*. *alba*	n/a	n/a	n/a	0.133	n/a	n/a	Vendramin *et al*., 1999
*A*. *nordmanniana*	n/a	n/a	n/a	0.021	n/a	n/a	Hansen *et al*., 2005
*A*. *cephalonica*	n/a	n/a	n/a	0.012	n/a	n/a	Parducci *et al*., 2001
*A*. *sibirica*	n/a	n/a	n/a	0.045	n/a	n/a	Semerikova *et al*., 2001
*A*. *nephrolepis*	n/a	n/a	n/a	0.009	n/a	n/a	Semerikova *et al*., 2001
*A*. *sachalensis*	0.630	n/a	n/a	0.010	n/a	n/a	Semerikova *et al*., 2001
*A*. *flinckii*	1	n/a	n/a	n/a	n/a	n/a	Jaramillo-Correa *et al*., 2008
*A*. *guatemalensis*	0.807	n/a	n/a	n/a	n/a	n/a	Jaramillo-Correa *et al*., 2008
*A*. *hickelii*	0.778	n/a	n/a	n/a	n/a	n/a	Jaramillo-Correa *et al*., 2008
*A*. *religiosa*	1	n/a	n/a	n/a	n/a	n/a	Jaramillo-Correa *et al*., 2008
***Picea***							
*P*. *mariana*	0.671	0.618	0.537	0.075	0.017	0.459	Jaramillo-Correa *et al*., 2004; Gérardi *et al*., 2010
*P*. *glauca*	n/a	n/a	n/a	0.028	0.028	n/a	Gérardi & Bousquet, unpublished
***Pinus***							
*P*. *banksiana*	0.569	0.307	n/a	0.043	0.043	n/a	Godbout *et al*., 2005, 2010
***Tsuga***							
*T*. *canadensis*	n/a	n/a	n/a	0.020	0.020	n/a	Lemieux *et al*., 2011

^1^ *F*_ST_, differentiation among populations;

^2^ *F*_SC_, differentiation among populations within groups;

^3^ *D*, Jost’s differentiation index;

^4^n/a, data not available.

Population differentiation within mtDNA BAPS groups was also significant but lower in *A*. *balsamea* than in the two largely sympatric conifer *P*. *banksiana* and *P*. *mariana* (Tables 2 and 3), indicating that balsam fir likely experience higher gene flow by seeds than most other boreal conifers. This translated into a higher rate of seed migration per generation than in other sympatric conifers (*N*_e_ *m*_mt_ = 1.77, 1.13 and 0.31 for *A*. *balsamea*, *P*. *banksiana* and *P*. *mariana*, respectively, data from Godbout *et al*., 2010 [7] and Gérardi *et al*., 2010 [6] for the last two species). However, mtDNA population structure remained strong, as evidenced by the presence of a phylogeographic structure (*N*_STmt_ > *G*_STmt_; *P* < 0.01).

Conversely, cpDNA differentiation among balsam fir populations (*G*_STcp_ = 0.104) was much higher than that of other boreal conifers with similar ranges such as *P*. *banksiana* [7], *T*. *canadensis* [33], *P*. *glauca* (Gérardi & Bousquet, unpublished data), except for *P*. *mariana* [6] (Table 3). However, when estimated using *P*. *mariana* populations occurring within the sampled range of *A*. *balsamea* (*i*.*e*. excluding populations from Alberta and westward), differentiation was much lower than that observed for *A*. *balsamea* (*G*_STcp_ = 0.0746 for *P*. *mariana*, estimated from Gérardi *et al*., 2010 [6]). Within the genus *Abies*, population differentiation is usually lower than that observed for balsam fir for cpSSR loci [69–71] or for nuclear microsatellites [73,74]. Only the widely distributed European fir *A*. *alba* shows comparable population differentiation estimate for cpSSRs [69] (Table 3). Thus, it is possible that fragmentation of such a large range into many geographically remote refugia during the LGM have contributed to increase the level of cpDNA differentiation in these two largely distributed species [1].

The *G*_STcp_ value was also much lower than Jost’s differentiation estimate (Table 3), likely due to *G*_ST_ dependency on within–populations genetic diversity [75–77]. Indeed, given that the cpSSR markers used in this study were highly polymorphic (*H*_cp_ = 0.773) and that only a few chlorotypes were shared among all populations, Jost’s differentiation index provides a more valid framework to estimate population differentiation [54,78]. Contrary to mtDNA, population differentiation within cpDNA BAPS groups was significant and much higher in *A*. *balsamea* (*F*_SCcp_ = 0.075; Table 2) than in *P*. *mariana* (Table 3) for the same type of SSR markers. Population differentiation within groups was also higher in *A*. *balsamea* than in *P*. *banksiana*, *P*. *glauca* and *T*. *canadensis* (Table 3). In these three species, *F*_SCcp_ and *F*_STcp_ were equivalent since no population structure was detected using cpSSRs, as in the present study. These results suggest that *A*. *balsamea* experiences substantially less pollen gene flow than any other sympatric North American conifer for which data were available. With 6.2 effective migrants per generation for cpDNA, balsam fir presented the lowest seed-plus-pollen migration rate of all species above-mentioned (*N*_e_ *m*_cp_ = 29.8, 24.5 and 11.1 for *P*. *mariana*, *T*. *canadensis* and *P*. *banksiana*, respectively). Low pollen-mediated gene flow is likely the main cause for the detection of a cpDNA phylogeographic structure in *A*. *balsamea* (*G*_STcp_ < *R*_STcp_, *P* < 0.01), contrary to what was observed in all other sympatric North American conifers surveyed.

Taken together, these evidences indicate that balsam fir populations are more differentiated and structured than those of any other conifer occurring in the same geographic area for which similar data are available. The most unexpected result, with regard to empirical observations in conifers, lies in the comparison between mtDNA and cpDNA differentiation. Indeed, Jost’s differentiation index showed that cpDNA differentiation was substantially higher than mtDNA differentiation in balsam fir (*D*_mt_ = 0.202 < *D*_cp_ = 0.610). Contrary to *G*_ST_, Jost’s *D* is independent of gene diversity [54,55], and therefore, provides a valid framework to compare genetic differentiation estimated from DNA markers with different levels of polymorphism and mutation rates (such as mtDNA sequence indels and cpDNA microsatellites, as used herein). Although gene flow through seeds remains substantially lower than that from seed-plus-pollen migration in balsam fir (*N*_e_ *m*_mt_ = 1.77 and *N*_e_ *m*_cp_ = 6.20), differentiation and migration estimates indicate that the homogenizing effect of pollen gene flow is considerably reduced in this species. This is further supported by estimates of differentiation among BAPS mtDNA and cpDNA groups, which were comparable (*D*_mt(group)_ = 0.552 and *D*_cp(group)_ = 0.594). Hence, balsam fir cpDNA should provide particularly valuable insights into the species’ postglacial history. It should be much more informative than seen in other sympatric conifers previously surveyed.

### Putative causes for reduced pollen gene flow and high cpDNA differentiation in balsam fir

Pollen of the genus *Abies* is very scarce in the fossil record [17,18]. This trend could be explained by poor pollen dispersal and/or relatively low production of pollen grains [17]. Although not obvious, structural property of fir pollen grains may account for their restricted dispersal [42,43], their size being about twice that of most *Pinus* species [41], but comparable to that of spruces [42,43]. However, several studies on total velocity of Pinaceae pollen grain, which refers to the the speed at which particles descend in still air owing to gravitational effects [45], have shown that balsam fir pollen had the highest value among North American boreal conifers (9.7, 2.7 and 3.2 for *A*. *balsamea*, *P*. *glauca* and *P*. *mariana*, respectively [79–81]). Such high fall speed of pollen may limit inter-population gene flow and long-distance dispersal aver generations. Thus, it may explain the unusually high cpDNA differentiation noted among balsam fir populations and higher propensity for cpDNA phylogeographical signatures to be conserved over longer time periods than in other conifers. A similar explanation was proposed for the widely distributed European fir *Abies alba*, a species also characterized by large pollen grains and for which high cpDNA population differentiation was reported [69] (Table 3).

An alternative hypothesis for high cpDNA differentiation can also be proposed. It would be related to Balsam fir’s sensitivity to natural disturbances such as fire, wind throw and insect pests [16]. More specifically, the species is the main host for the spruce budworm (*Choristoneura fumiferana* (Clemens)) [82], an indigenous lepidopteron which has been an important and recurrent destructor of *A*. *balsamea* and *P*. *glauca* populations in central and eastern Canada [83,84]. Budworm larvae destroy, among others, female and male floral buds [85], causing a drastic decrease of the balsam fir reproduction capacity [82,86]. This insect pest causes recurrent and considerable damages and mortality in balsam fir stands [87], but is also essential for their natural regeneration [88]. Major outbreaks are estimated to occur every 35 years on average [82,89,90], which would roughly represent one balsam fir generation (average sexual maturity reached at around 25 years [41]). During these major outbreaks, balsam fir mortality can reach 91%, while *P*. *glauca* is generally less affected (52% [91]). Thus, the fact that balsam fir may not be able to reach its full reproductive potential between two outbreaks could also account for reduced gene flow by pollen and increased congruence between mtDNA and cpDNA population structures. This hypothesis is further supported by fossil data, which indicate that spruce budworm maintained a stable presence in Québec since 8 ky, with intense periods of activity ([92]). Although noteworthy, such putative influence of the recurrence of an insect pest on the long-term demography and reproductive effort of a conifer species remains to be formally tested.

### Delineation of glacial lineages in balsam fir

**Fig 5:**
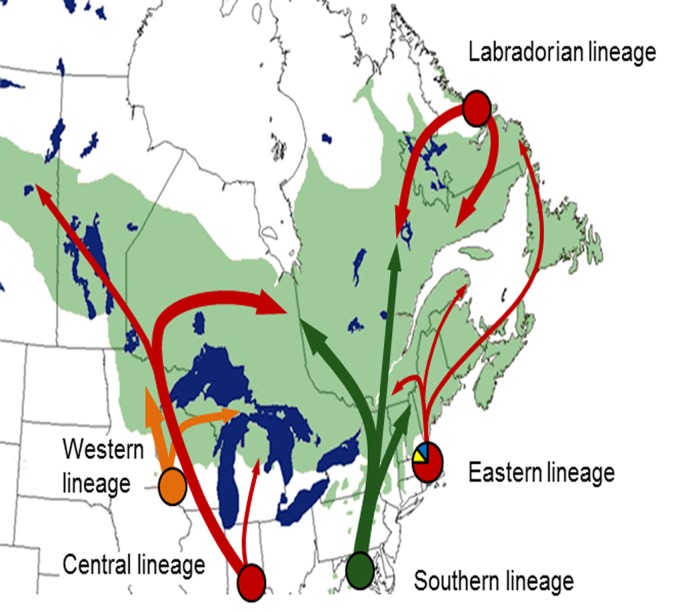
Summary of inferred phylogeographical processes that led to the current distribution of mtDNA and cpDNA diversity in *Abies balsamea*. Putative glacial refugia (circles) and postglacial recolonization routes (full arrows) are indicated.

Overall, five genetically distinct glacial refugia were inferred from mtDNA and cpDNA variation in balsam fir (Fig 5). The first mtDNA group was composed of populations carrying almost exclusively mitotype I but it was divided in two geographically disjunct subgroups. Mitotype I is likely an ancient mitotype, given that it was fixed in *A*. *fraseri* (Fig 3). The western population subset of *A*. *balsamea* (Saskatchewan, Ontario, Manitoba) was geographically isolated from the eastern subset (northern New-England, northeastern Québec, Newfoundland and Labrador) by a large area where populations carried predominantly mitotype II, along with mitotype I (mtDNA group #2). Hence, these two disjunct population subsets do not likely originate from the same glacial refugium despite their similarity in mtDNA composition. This hypothesis is in line with the cpDNA population structure, which showed a clear genetic divergence between populations from northeastern Canada (Labrador, Newfoundland and the Maritimes, cpDNA group #4), and western Canada (cpDNA group #1), corresponding approximately to these two disjunct subgroups of mtDNA group #1. Given the very low polymorphism observed in the mtDNA of balsam fir and low mutation rate of plant mtDNA [65], it is possible that these two geographically distinct glacial populations did not have enough time to evolve distinctive mtDNA polymorphisms, contrary to the sampled cpDNA microsatellites which are characterized by much higher mutation rates in conifers [93].

The occurrence of mitotypes I and III in the Great Lakes region likely represents two genetically distinct *A*. *balsamea* lineages originating from two geographically distinct glacial refugia located south of the continental glacier. A first lineage (Central lineage, Fig 5), essentially carrying mitotype I, would correspond to the western population subset of mtDNA group #1. These populations may have originated from a large glacial population located south of the Great Lakes, as previously proposed for *P*. *banksiana* [7,20] and for *P*. *mariana* [6,19]. The fixation of mitotype I in population located northwest of the Great Lakes (northern Ontario, Manitoba and Saskatchewan, Fig 3) indicates that most migrant that colonized this region came from this refugium. A second lineage (Western lineage, Fig 5), mainly composed of individuals carrying mitotype III, would have persisted in a cryptic refugium presumably located west of the Great Lakes, at the very margin of the Laurentide ice sheet. The co-occurrence of mitotype I and III in most populations from the Western Great Lakes (mtDNA group #2) is in line with this idea. A possible location for the cryptic refugium would be the ‘Driftless Area’, in the south of the states of Wisconsin, Minnesota and northern Iowa, as previously hypothesized for *Fagus grandifolia* [94], *Quercus sp*. [95] and *Acer rubrum* [96]. In agreement with this hypothesis, the first occurrence of *Abies* fossil pollen at the margin of the Laurentide ice sheet in these states was recorded at 15ka [97–99], which corresponds to the early deglaciation phase in the region. However, the absence of mitotype III further north suggests that such cryptic refugium was likely of limited size and that the contact between these two glacial lineages occurred in the early stage of the colonization process. This hypothesis is supported by fossil data, which indicate that populations from the south Great Lakes region expanded rapidly soon after the LGM [17], as evidenced by a significant increase of *Abies* pollen between 15ka and 12ka. It is also supported by cpDNA data, which revealed the occurrence of two genetically distinct lineages in western Canada. The first cpDNA lineage, which included the westernmost populations (Minnesota, Manitoba and Saskatchewan), would correspond to the mtDNA lineage originating from the cryptic refugium (Western lineage, Fig 5), while populations surrounding the Great Lakes (Ontario, Michigan and Wisconsin) would carry the genetic signature of the main glacial population located south of the Great Lakes (Central lineage). Alternatively, this mtDNA pattern could result from allele surfing during postglacial population expansion [100,101]. Accordingly, western North America would have been colonized by a single lineage made of individuals carrying mitotypes I and III. However, this explanation appears less plausible than the previous one, given that cpDNA data provided support for the persistence of two lineages in this region and that the frequency of mitotype I did not increase gradually along the inferred migration route, as would be expected with allele surfing [100,102]. Finally, concordance between the present-day geography of mtDNA and cpDNA lineages was not complete, presumably reflecting differential gene flow between the two cytoplasmic genomes and the fact that cytoplasmic capture took place during the colonization process (see below the section on cytoplasmic capture).

Despite its high mtDNA homogeneity, the eastern population subset of mtDNA group #1 likely represents two distinct glacial lineages. The first lineage (Labradorian lineage, Fig 5) would encompass populations from Labrador, and possibly those from northern Quebec. These populations, which carry mitotype I almost exclusively, would have persisted in a refugium located in the Labrador region. Such a refugium was previously proposed for *P*. *mariana* [19] or for the migratory songbird *S*. *ruticilla* [23], among others. This hypothesis is further supported by cpDNA evidence, which showed that populations from Labrador and northern Quebec (cpDNA group #4) were genetically distinct from adjacent populations (Fig 4). This population subset also carried the highest diversity of all cpDNA groups and a large number of rare alleles (frequency lower than 1%, S1 Table). The cpDNA dendrogram also showed that populations from Labrador presented genetic similarities with the outgroup *A*. *fraseri*, thus highlighting their genetic uniqueness and the likely ancestral nature of their polymorphism (Fig 4). The remaining populations, located in Newfoundland, the Maritimes and southern Quebec would belong to a different glacial lineage. This lineage (Eastern lineage, Fig 5) may also encompass populations from mtDNA group #4, which carry a unique genetic background (predominance of mitotypes IV and V). These populations would have either originated from a coastal refugium located in the Maritimes or the coastal areas of northern Maine, as previously proposed for pines (*P*. *resinosa* [28]; *P*. *banksiana* [7]), or from a cryptic refugium putatively located east of the Appalachians at the margin of the continental glacier (eastern lineage, Fig 5), where habitats suitable to balsam fir were more likely to be found. This hypothesis is further supported by the remote record of *Abies* pollen around 15ka ago in the northern Appalachians [17]. As evidenced by mtDNA data, these populations would have first expanded northward into the St. Lawrence River valley and then eastwards into the Maritimes and Newfoundland. Such refugium location and colonization pathway was previously proposed for *P*. *mariana* [6, 19], where populations from southern Quebec, the Maritimes and Newfoundland belonged to a genetically distinct and diverse mtDNA lineage. However, contrary to *P*. *mariana*, this lineage was surrounded by populations carrying another widespread and frequent mitotype (mitotype II). These populations, which correspond to mtDNA BAPS group #3 (Fig 3), would form a distinct lineage (Southern lineage, Fig 5), possibly originating from a refugium located further south in the southern Appalachians. This region was designated as a major refugium area for several North American tree species (reviewed in [1]), including for the conifer *T*. *canadensis* [22], and possibly represented the region of origin for *Picea rubens* during the Pleistocene [103]. The current distribution of this *A*. *balsamea* lineage suggests that, although the species persisted in a southern refugium, migrants expanded northward in great numbers and early enough to prevent the Eastern lineage to migrate westward and northward into central and northern Quebec (Fig 5), a pattern previously observed in *P*. *banksiana* [7,20]. In line with this hypothesis, the fossil record indicates that, while pollen density remained rather stable between 15ka and 12ka ago in the northern Appalachians, a major increase in fir pollen was observed along the whole Appalachian range further south [17]. Around 12ka ago, low pollen concentration was recorded along the whole margin of the ice sheet between Lake Michigan and the Atlantic coast, suggesting that both lineages completed their northward migration as far as they could. At this point, the Eastern lineage likely occupied all northeastern deglaciated terrain (Maritimes and the St. Lawrence Valley) and was surrounded in the south (New England) and west (southeastern Ontario) by the Southern lineage (Fig 5).

### Cytoplasmic capture

Evidence of cytoplasmic capture was detected in several parts of *A*. *balsamea* range due to differential gene flow between cytoplasmic genomes, as illustrated by the observed geographical discordances between mtDNA and cpDNA lineages. Populations from western Canada, Wisconsin and the Upper Michigan Peninsula harboured an mtDNA signature typical of the Western lineage and a cpDNA signature typical of the Central lineage (Figs 3 and 4). While this region is likely a zone of contact between these two intraspecific lineages, as suggested by the co-occurrence of mitotype I and III in these populations, the predominance of mitotype I may indicate that individuals from the Central lineage are currently predominant. This would explain why populations are carrying a cpDNA background representative of the Central lineage in this region. The case of the westernmost populations (Manitoba and Saskatchewan), that carried a Central mtDNA lineage and a Western cpDNA lineage, is however more interesting. Evidence from deglaciation patterns and the fossil record, combined with the observation of restricted gene flow by pollen in balsam fir, suggests that cytoplasmic capture occurred in the early colonization stages. According to this scenario, the native cpDNA of the first migrants from the Central lineage that reached the margin of the ice sheet would have been replaced by that of the more abundant individuals from the Western lineage that persisted locally during the LGM. Migrants carrying this mixed cytoplasmic background would have then spread northwestward into Manitoba and Saskatchewan, while migrants from the Central lineage would have gradually outnumbered those from the Western lineage at the trailing edge of the migration front, and spread eastward into the north of the Great Lakes later on as the ice receded.

Although being a less parsimonious explanation, due to evidence for limited pollen dispersal in balsam fir, cytoplasmic capture may have also occurred after the initial colonization of Manitoba and Saskatchewan by individuals from the Western lineage. Under such a scenario, the native cpDNA of individuals from the Western lineage would have been gradually replaced by that of individuals from the Central lineage during the Holocene.

The analysis of cpDNA variation also revealed that, with the exception of populations from Labrador and northern Quebec, all populations from southeastern Canada carried the cpDNA background of the Southern lineage (Fig 4). This observation indicates that the native cpDNA of the Eastern lineage would have been completely replaced by that of the Southern lineage as a result of pollen gene flow. It has been hypothesized that prevailing westerly winds since the LGM [104] would have largely promoted unidirectional eastward pollen gene flow, and ultimately, would be responsible for the replacement of native cpDNA of eastern lineages by that of western lineages. However, since pollen gene flow appears restricted in *A*. *balsamea*, it is also possible that cytoplasmic capture occurred at the beginning of the colonization process, when the Eastern and Southern lineages first came in contact, as was hypothesized above for western Canada. This inference is supported by the fact that an additional cpDNA lineage encompassing populations from Newfoundland and eastern Maritimes was detected in the original BAPS partition (cpDNA group 5, S1 Fig). Under this scenario, cytoplasmic capture would have taken place in populations from eastern Maritimes and Quebec only.

While both scenarios have likely contributed to such cytoplasmic capture pattern, the last scenario may explain why the Maritimes and Quebec is the only geographic area where balsam fir cpDNA lineages extended further eastward than their mtDNA counterparts, as typically observed in North American boreal conifers [6,7]. This may also explain that several populations from northern Quebec carry the cpDNA background typical of the Labrador lineage and the mtDNA background typical of the Central lineage (Figs 3 and 4). Indeed, these populations were likely grouped with the Central lineage by the present spatial analyses because they carried mitotype II (characteristic of the Central lineage) at various frequencies, along with mitotype I, the only mitotype found in populations from the Labrador lineage. Thus, this mixed mtDNA background may also be considered as a suture zone between these two intraspecific lineages. Under such a scenario, the fact that populations from this hypothetical suture zone carry the cpDNA background of the Labrador lineage may indicate that migrants from this lineage out-numbered those from the Central lineage when the contact occurred.

Such widespread phenomena of mitochondrial genome capture and new inter-mixed cytoplasmic genomic background from different glacial lineages is not unique to *A*. *balsamea* but rather appears to be the rule at the intraspecific level for geographically widespread conifers in which genetically distinct glacial lineages are still detectable. Such phenomena of mtDNA genome capture have been observed between intraspecific glacial lineages in *P*. *mariana* at various places across Canada [6], in *P*. *banksiana* in eastern Canada [7], and in *P*. *menziesii* between coastal and interior varieties in western North America [10]. Cytoplasmic capture has also been increasingly observed at the interspecific level, for instance in the *Picea asperata* complex in China [105], in the *A*. *nephrolepis—A*. *sachalinensis* complex [72] or between *P*. *banksiana* and *P*. *contorta* in their zone of contact and beyond in western and central Canada [106].

## Conclusion

The present study reinforces the view that genetic signature of historical processes such as vicariance or demographic fluctuations on phylogeographic patterns can be greatly influenced by species-specific morphological traits and life history. Indeed, cpDNA gene flow appeared limited in balsam fir, presumably owing to its particularly low pollen production and dispersal, and to the potential negative impact of recurrent spruce budworm outbreaks on the reproductive effort of balsam fir. As a result, concordance between cpDNA and mtDNA lineages was higher in this species than in any other largely distributed North American conifers, which prompted new hypotheses about the cytoplasmic capture process. To date, mtDNA capture in North American conifers was hypothesized to have occurred via long-distance pollen gene flow after the colonization of deglaciated terrain by genetically distinct lineages (*e*.*g*. [6]). Conversely, mtDNA capture events observed in balsam fir are thought to have occurred mainly at the early beginning of postglacial recolonization, when lineages came in contact at the margin of the ice front. For the time being, the adaptive implications arising for the existence of these multiple suture zones and inter-mixing of genomic compositions between previously isolated lineages are unknown. Overall, genetic diversity will likely be increased in these regions, as previously suggested for other boreal conifers (*e*.*g*. [20]). A detailed analysis of candidate genes or candidate SNPs involved in adaptive mechanisms would shed light on potentially divergent selection between glacial lineages (*e*.*g*. [56]) and to better grasp the implications of the existence of these zones at the ecological and functional levels. In the meanwhile, the cautionary principle would dictate to integrate these findings in the management and conservation of natural genetic resources of balsam fir.

In addition, despite the generally high dispersal potential of conifer pollen, the strong structure of the cpDNA diversity observed in balsam fir points to a rare case of restricted pollen gene flow in conifers. It also indicates that fragmentation at the landscape and natural range levels in balsam fir can turn out to bear more genetic consequences than in other Pinaceae, especially when distant populations are considered. Implications for gene conservation in this species and other ones with similar life history and reproductive features should be further studied. From a biogeographic perspective, this study also brought support for the existence of three controversial refugia, in the Driftless area of central United States, in Labrador region, and in the Maritimes-Appalachians region of eastern Canada and northeastern United States. However, regional studies with increased sampling would help deciphering the exact location of these refugia and guide conservation efforts by assessing their relative significance in term of genetic diversity and differential of adaptation, for instance to coastal versus more continental climates.

## Supporting Information

S1 Fig Logarithm relationship between the number of groups (*k*-value) and the log(lm) value for BAPS analysis of (a) mtDNA and (b) cpDNA spatial structures of *Abies balsamea*. Arrow shows the inflection point and the partition selected. See the section Results for more information.
(TIF)

S2 Fig (a) Spatial distribution of BAPS initial mtDNA groups (optimal partition, *k* = 7 corresponding to the seven colored tracings on the map). (b) Neighbor-Joining dendrogram based on chord genetic distances among BAPS groups; the color of filled circles matches the color of BAPS groups on the map; putative suture zones are indicated by a square; ellipses correspond to the final grouping presented in Fig 3.
(DOCX)

S3 Fig (a) Spatial distribution of BAPS initial cpDNA groups (optimal partition, *k* = 9 corresponding to the nine colored tracings on the map). (b) Neighbor-Joining dendrogram based on chord genetic distances among BAPS groups; the color of filled circles matches the color of BAPS groups on the map; putative suture zones are indicated by a square; ellipses correspond to the final grouping presented in Fig 4; initial BAPS group #3 was redistributed into two consolidated groups following arrows, at a rate of 12 individuals attributed to the green ellipse and 11 individuals attributed to the blue ellipse, based on their chlorotypes.
(DOCX)

S1 Table Detailed populations’ geographic and genetic data. Table S1 contains three sheets corresponding to geographic data, mtDNA-cpDNA haplotype counts and genetic diversity indices, respectively.
(XLSX)

S2 Table Target regions, sequences, annealing temperatures and expected size of PCR products for primer pairs used to amplify mtDNA regions of *Abies balsamea*. (DOCX)
