# Supplementary figures and images for: Less Pollen-Mediated Gene Flow for More Signatures of Glacial Lineages: Congruent Evidence from Balsam Fir cpDNA and mtDNA for Multiple Refugia in Eastern and Central North America
Source: PLoS One. 2015 Apr 7;10(4):e0122815. doi: 10.1371/journal.pone.0122815 (PMC4388536; doi:10.1371/journal.pone.0122815)

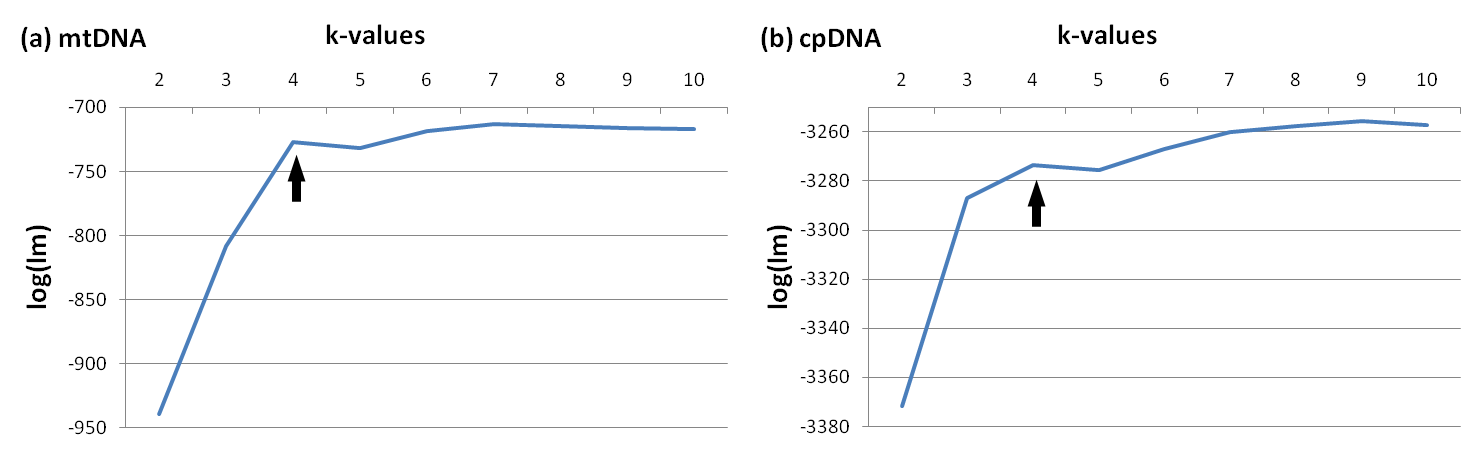

Supplement: S1 Fig — Arrow shows the inflection point and the partition selected. See the section Results for more information. (TIF) [file pone.0122815.s001.tif]
