# Supplementary material for: Less Pollen-Mediated Gene Flow for More Signatures of Glacial Lineages: Congruent Evidence from Balsam Fir cpDNA and mtDNA for Multiple Refugia in Eastern and Central North America
Source: PLoS One. 2015 Apr 7;10(4):e0122815. doi: 10.1371/journal.pone.0122815 (PMC4388536; doi:10.1371/journal.pone.0122815)
Supplement: S2 Fig — (a) Spatial distribution of BAPS initial mtDNA groups (optimal partition, k = 7 corresponding to the seven colored tracings on the map). (b) Neighbor-Joining dendrogram based on chord genetic distances among BAPS groups; the color of filled circles matches the color of BAPS groups on the map; putative suture zones are indicated by a square; ellipses correspond to the final grouping presented in Fig 3. (DOCX) [file pone.0122815.s002.docx]

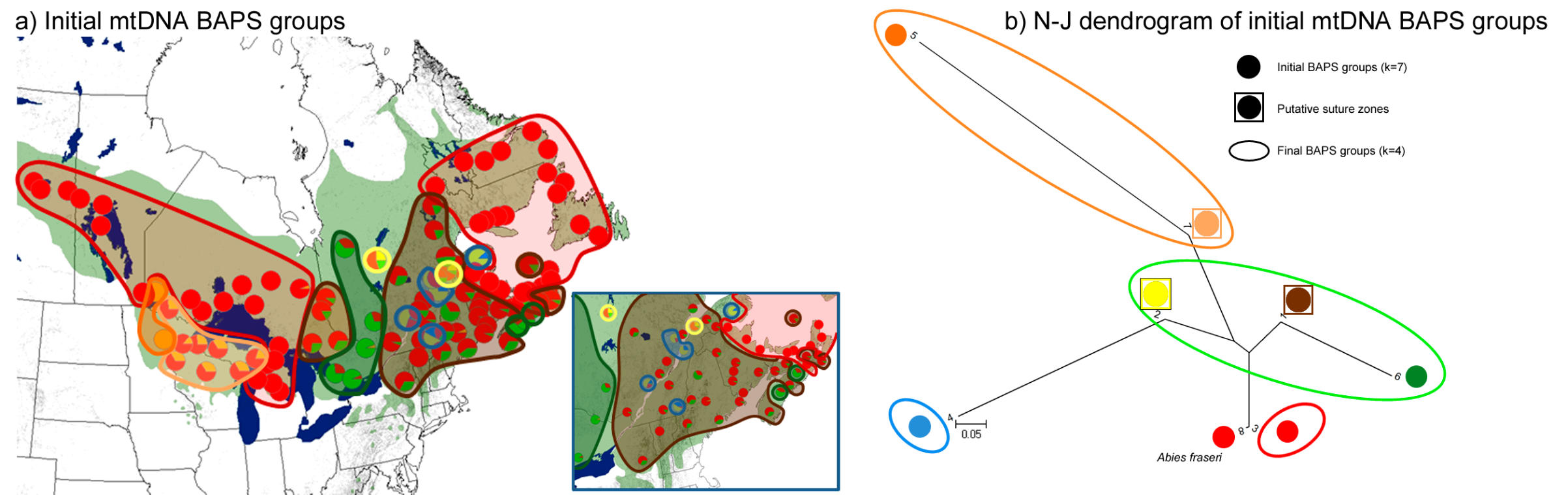


Note for S2 Figure

The initial mtDNA BAPS grouping yielded seven groups of populations genetically distinct and spatially structured (Fig S4a). The mtDNA dendrogram illustrates that the four initial mtDNA groups, which were assumed to be most representative of ancestral lineages in the final grouping (mtDNA groups 3, 4, 5 and 6), were also the most genetically divergent (Fig. S4b). Each of these four groups carried a specific mtDNA background (mitotype I for group 3, mitotypes IV and V for group 4, mitotype III for group 5 and mitotype II for group 6). Accordingly, the three remaining initial groups (mtDNA groups 1, 2 and 7) occupied intermediate positions in the dendrogram and carried mixed mtDNA backgrounds representative of different lineages. The geographic location of these last three groups was also consistent with the view that they represented modern suture zones between glacial lineages, hence providing support for the final grouping. The genetic background of populations from group 7 (mitotype I and III) suggested that this group is a suture zone between groups 3 and 5. Group 7 and 5 were likely merged in the final grouping based on their spatial proximity and the narrow spatial distribution of mitotype III (Fig S4a). Group 1 was merged with group 6 in the final grouping. Group 1 included populations carrying mitotype I and II, two variants representative of groups 3 and 6, respectively. However, group 1 is made of two geographically disjunct subgroups spatially surrounding group 6, which suggests that this group represent a suture zone between group 6 and adjacent lineages (Fig S4a). Finally, group 2 included two populations, each of them carrying three mitotypes (representative of groups 3, 4 and 6). This suggests that these populations represent a suture zone between three lineages (Fig S4a). In the final grouping, group 2 was merged with group 6 based on genetic affinities. However this group collapse had little impact on the large-scale mtDNA genetic pattern given the very limited number of populations involved.
