# Supplementary material for: Less Pollen-Mediated Gene Flow for More Signatures of Glacial Lineages: Congruent Evidence from Balsam Fir cpDNA and mtDNA for Multiple Refugia in Eastern and Central North America
Source: PLoS One. 2015 Apr 7;10(4):e0122815. doi: 10.1371/journal.pone.0122815 (PMC4388536; doi:10.1371/journal.pone.0122815)
Supplement: S3 Fig — (a) Spatial distribution of BAPS initial cpDNA groups (optimal partition, k = 9 corresponding to the nine colored tracings on the map). (b) Neighbor-Joining dendrogram based on chord genetic distances among BAPS groups; the color of filled circles matches the color of BAPS groups on the map; putative suture zones are indicated by a square; ellipses correspond to the final grouping presented in Fig 4; initial BAPS group #3 was redistributed into two consolidated groups following arrows, at a rate of 12 individuals attributed to the green ellipse and 11 individuals attributed to the blue ellipse, based on their chlorotypes. (DOCX) [file pone.0122815.s003.docx]

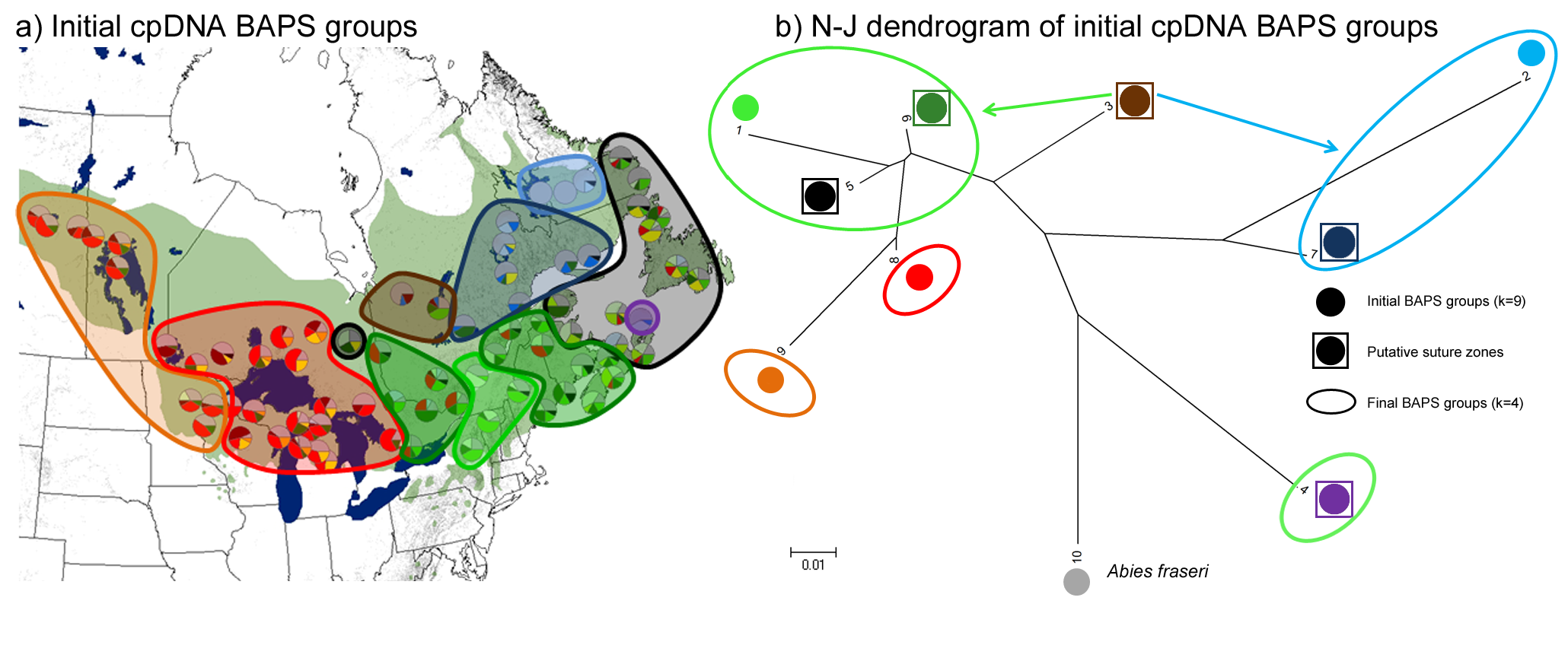


Note for S3 Figure

The initial cpDNA BAPS grouping yielded nine groups of populations genetically distinct and spatially structured (Fig S5a). Although more complex than for mtDNA, genetic relationships among BAPS initial cpDNA groups also supported the view that several suture zones were originally identified as distinct groups. The cpDNA dendrogram illustrates that, with the possible exception of group 8, the four initial cpDNA groups, which were assumed to be most representative of ancestral lineages in the final grouping (cpDNA groups 1, 2, 8 and 9), were also the most genetically divergent (Fig. S5b). In the final grouping, groups 2 and 7 were merged based on their low genetic divergence and spatial proximity (forming the Labradorian lineage, Fig 5) (Fig S5a). Groups 5 and 6 were equally genetically distant from group 1 (Southern lineage, Fig 5) than from group 8 (Western lineage, Fig 5) (Fig S5b). However, they were merged with group 1 due to spatial proximity between groups 1, 5 and 6 (forming the Southern lineage, Fig 5). The spatial arrangement of groups 1 and 6 was analogous to that of mtDNA groups 1 and 6 (see Fig S4a). Group 6 was made of two spatially disjunct subgroups surrounding group 1, suggesting that group 6 represented a suture zone between group 1 and adjacent lineages (Fig S5a). The situation of group 5 was however more ambiguous. While this group may represent a suture zone between the Southern lineage and the Labradorian lineage, its spatial distribution at the easternmost part of the species’ range (mainly in the Maritimes and Newfoundland) also suggests that it could represent a distinct lineage. This hypothesis was further supported by mtDNA evidence for the persistence of a distinct lineage in this region (Fig 5, lines 520-551). Both hypotheses were discussed in the manuscript since they appeared equally likely (lines 579-592). Group 3, which included 2 populations, was likely a suture zone between the Labradorian lineage and the Southern lineage according to its spatial location. In the final grouping, one population was merged with the Labradorian lineage, while the remaining one was merged with the Southern lineage. Finally, group 4, which included a single population, was merged with the eastern lineage. This group was not well supported by BAPS, as illustrated by his extremely high assignment uncertainty. According to BAPS output, assigning this population to any group, other than the two westernmost ones (groups 8 and 9), would have had no impact on the likelihood associated to the population partition.
