# Supplementary material for: Less Pollen-Mediated Gene Flow for More Signatures of Glacial Lineages: Congruent Evidence from Balsam Fir cpDNA and mtDNA for Multiple Refugia in Eastern and Central North America
Source: PLoS One. 2015 Apr 7;10(4):e0122815. doi: 10.1371/journal.pone.0122815 (PMC4388536; doi:10.1371/journal.pone.0122815)
Supplement: S2 Table — (DOCX) [file pone.0122815.s005.docx]

**Table S2.** Target regions, sequences, annealing temperatures and expected size of PCR products for primer pairs used to amplify mtDNA regions from the balsam fir panel. See Material and Methods for more panel description.

| Genomic region | Sequence of Forward primer  (5’-….-3’) | Sequence of Reverse primer  (5’-….-3’) | Primer source |
| --- | --- | --- | --- |
| *ccb*203 | ASGTTCTACGGACCGATGCC | CACGGGGAGGGAGCRGGCGA | Duminil *et al.,* 2002 |
| *ccb*256 | GGAAGTTAGCAAAGTTAGAC | TTGTTCTTAACAGCGATGGC | Duminil *et al.,* 2002 |
| *coxI* | TTATTATCACTTCCGGTACT | AGCATCTGGATAATCTGG | Lu *et al.,* 1998 |
| *cox*2 (intron 1) | TTTTCTTCCTCATTCTKATTT | CCACTCTATTGTCCACTTCTA | Duminil *et al.,* 2002 |
| *cox*2 (intron 2) | TAGRAACAGCTTCTACGACG | GRGTTTACTATGGTCAGTGC | Duminil *et al.,* 2002 |
| *cox*3 | GTAGATCCAAGTCCATGGCCT | GCAGCTGCTTCAAAGCC | Wu *et al.*, 1998 |
| *matR* | CGACAGAAGCACGAAATTCC | ACCCGACGATAACTAGCTTC | Jaramillo-C *et al.,* 2003 |
| *mh02* | TTTTAGGGCCATTTGCCTGC | TCTATGGACAAGAGCCCGACCT | Jeandroz *et al.,* 2002 |
| *mh05* | GGGAGTCAGCGAAAGAAGTAAG | AGTCTCAGAGCCAGAAGCAG | Jeandroz *et al.,* 2002 |
| *mh08* | GTCATCCCTATCTCCTGGAC | CTAGTAGGATTAAGTGGCAACC | Jeandroz *et al.,* 2002 |
| *mh09* | TCATCCATCCTCCAGCAACA | TCATCCCCAGAAAGAGACAG | Jeandroz *et al.,* 2002 |
| *mh09’* | CCATCCAGCCATGTCTCATC | AGGGCTTCACATAGAGCATC | Jeandroz *et al.,* 2002 |
| *mh10* ^a^ | CACTGCTCACCTTCACATTC | CTTCACATAGAGCATCGATCAC | Jeandroz *et al.,* 2002 |
| *mh27* | TGCTTTCCAATTTACCACGAG | GATACGCTTTCCTGGCATAC | Jeandroz *et al.,* 2002 |
| *mh33* | TTCCCCAGACAGAACAGATAG | GCTCTTAAGTGCTGGTTGATG | Jeandroz *et al.,* 2002 |
| *mh33’* | CGAAGGAAGGAATGAAGGTG | GCTCTTAAGTGCTGGTTGATG | Jeandroz *et al.,* 2002 |
| *mh34* | TTGGATCACCCACTTCCT | TAAGCACACCTCTGCATCC | Jeandroz *et al.,* 2002 |
| *mh35* | CGATGACATCTCTTAGCTTCC | TGGGGAATAGGATTCGGGTAAG | Jeandroz *et al.,* 2002 |
| *mh38* | CCGTCCCCTATCCATCAAAC | CCCTGAGCGAGATTGAATTAG | Jeandroz *et al.,* 2002 |
| *mh44* | ATGACTGGAAGAATTGCTCAC | TTCACTTGATACTCACCCCC | Jeandroz *et al.,* 2002 |
| *mh50* | AGAATGGCAGCAACTAATAAGC | ACTATGCACTTCCCTCCCTC | Jeandroz *et al.,* 2002 |
| *mp6* ^b^ | CGCTTCACTCTAACCCTTCC | CCTTCACTCTTACAAACGCC | Jaramillo-C *et al.,* 2003 |
| *nad1* (intron b/c) | GCATTACGATCTGCAGCTCA | GGAGCTCGATTAGTTTCTGC | Demesure *et al.,* 1995 |
| *nad3*-*rps12* (i.r.)^c^ | CAGAAGTCGTTTCGATATACG | TTTCTCCGAAGCTCGGGTACG | Soranzo *et al.,* 1999 |
| *nad3* (intron 1) | TTCCCCATGAATGGAAGAAG | ATTGATTCGATGTAGGCATCG | Soranzo *et al.,* 1999 |
| *nad4* (intron1) | ATACGATTGATTGGTCTGTG | TGAACTGGTACCATAGGCACTTT | Wu *et al.,* 1998 |
| *nad4* (intron2) | CTCCTCAGTAGCCCATATGA | AACCAGTCCATGACTTAACA | Duminil *et al.,* 2002 |
| *nad4*L-*orf25* (i.r.)^c^ | TATTACTTTCCGAGTCCGGGG | TCTTCTTCGAACTTGATGCAC | Kubo *et al.,* 2000 |
| *nad5* (intron 1)^d^ | AGTCCAATAGGGACAGCACAC | GCTTTGATAGCTGCTTTATCTGC | Jaramillo-C *et al.,* 2003 |
| *nad5* (intron 4) | ATAAGTCAACTTCAAAGTGGA | CATTGCAAAGGCATAATGAT | Wu *et al.,* 1998 |
| *nad5-*4*Ab* | ATCGATGGCCATGTCTATTA | AGTTAGGCTAGGGACAATGAC | This study |
| *nad7* (intron 1) | GGAACCGCATATTGGATCAC | GTTGTACCGTAAACCTGCTC | Jaramillo-C *et al.,* 2004 |
| *nad7* (intron 2) | GCTTTACCTTATTCTGATCG | TGTTCTTGGGCCATCATAGA | Duminil *et al.,* 2002 |
| *nad7* (intron 3) | TAGGATCCTGATCGAGCAAG | CTGGACAAGCTTTAGGGGAA | Bonen *et al.,* 1994 |
| *nad7* (intron 3) Alt. | TCTATGATGGCCCAAGAACA | ACACCAAATTCTCCTTTAGG | Duminil *et al.,* 2002 |
| *orf25* | AAGACCRCCAAGCYYTCTCG | TTGCTGCTATTCTATCTATT | Duminil *et al.,* 2002 |
| *rpl5* | AGTGGTAAAGTCTCATCT | ATYGTGTGAAATAAGAGTAG | Duminil *et al.,* 2002 |
| *rps3* (intron 1) | CCGAATCGTAGTTCAGATCCA | GTGCAACGCCTCTGACATA | Jaramillo-C *et al.,* 2006 |
| *rps3* (intron 2) | TTTGGCTTTCGTCTCGGTAG | CCCTCACTTCGTTTCGTTCT | Jaramillo-C *et al.,* 2006 |
| *rps14*-*cob* (i.r)^bc^ | CACGGGTCGCCCTCGTTCCG | GTGTGGAGGATATAGGTTGT | Demesure *et al.,* 1995 |
| SSU *rRNA* (V1 region) | GAGTTTGATCCTGGCTCAGA | AGTYGCAGTGTGGCTG | Duff and Nickrent 1999 |
| SSU *rRNA* (V7 region) | CTGCATGGCTGTCGTC | CCACCTTCCTCCAGT | Duff and Nickrent 1999 |
| *trn*H-mt | GATCCAATAGCGAGTATAGACGTG | AAAGGATTTGAAAACCACTCCTC | Maréchal-Drouard *et al.,* 1996 |

^a^ Targets the same region than *mh09*’ + 200 bp (up stream);

^b^ Mitochondrial plasmid-like DNA repeat region from *Picea abies*

^c^ i.r.: Intergenic region.

**References:**

Bonen L, Williams K, Bird S, Wood C (1994) The NADH dehydrogenase subunit 7 gene interrupted by four group II introns in the wheat mitochondrial genome. Mol Gen Genet 244: 81-89.

Demesure B, Sodzi N, Petit RJ (1995) A set of universal primers for amplification of polymorphic non-coding regions of mitochondrial and chloroplast DNA in plants. Mol Ecol 4: 129-131.

Duff RJ, Nickrent DL (1999) Phylogenetic relationships of land plants using mitochondrial small-subunit rDNA sequences. Am J Bot 86: 372-386.

Duminil J, Pemonge M-H, Petit RJ (2002) A set of 35 consensus primer pairs amplifying genes and introns of plant mitochondrial DNA. Mol Ecol Notes 2: 428-430.

Jaramillo-Correa JP, Bousquet J, Beaulieu J, Isabel N, Perron M, Bouillé M (2003) Cross-species amplification if mitochondrial DNA sequence-tagged site markers in conifers: the nature of polymorphism and variation within and between species in *Picea*. Theor Appl Genet 106: 1353-1367.

Jaramillo-Correa JP, Beaulieu J, Bousquet J (2004) Variation in mitochondrial DNA reveals multiple distant glacial refugia in black spruce (*Picea mariana*), a transcontinental North American conifer. Mol Ecol 13: 2735-2747.

Jaramillo-Correa JP, Bealieu J, Ledig FT, Bousquet J (2006) Decoupled mitochondrial and chloroplast DNA population structure reveals population Holocene collapse and population isolation in a threatened Mexican-endemic conifer. Mol. Ecol. 15: 2787-2800.

Jeandroz S, Bastien D, Chandelier A, Du Jardin P, Favre JM (2002) A set of primers for amplification of mitochondrial DNA in *Picea abies* and other conifer species. Mol Ecol Notes 2: 389-391.

Kubo T, Yamamoto MP, Mikami T (2000) The *nad4L-orf25* gene cluster is conserved and expressed in sugar beet mitochondria. Theor Appl Genet 100: 214-220.

Lu M-Z, Szmidt AE, Wang XR (1998) RNA editing in gymnosperms and its impact on the evolution of the mitochondrial *coxI* gene. Plant Mol Biol 37: 225-234.

Maréchal-Drouard L, Kumar R, Remacle C, Small I (1996) RNA editing of larch mitochondrial tRNA(His) precursors is a prerequisite for processing . Nucl Acid Res 24: 3229-3234. Ref Genbank: Z70031.

Soranzo N, Provan J, Powell W (1999) An example of microsatellite length variation in the mitochondrial genome of conifers. Genome 42: 158-161.

Wu J, Krutovskii KJ, Strauss SH (1998) Abundant mitochondrial genome diversity, population differentiation and convergent evolution in pines. Genetics 105: 1605-1614.
